# The potential of oral healthcare providers to recognise early systemic disease

**DOI:** 10.1038/s41415-025-8814-0

**Published:** 2025-08-22

**Authors:** Abdouldaim Ukwas, Stephen R. Porter

**Affiliations:** UCL Eastman Dental Institute, UCL Rockefeller Building, 21 University Street, London, WC1E 6DE, United Kingdom

## Abstract

Like many other body structures, the mouth is often affected by disease that principally arises from, or targets, several tissue sites. Indeed, systemic disorders can manifest on the oral mucosa as the first, only, or become the most severe manifestation of a systemic disease and/or become the most significant factor to adversely affect an individual's quality of life. Oral healthcare providers (dentists, therapists and hygienists) may be the first to observe an abnormality in the mouth which sometimes indicates a significant systemic disease unknown to the patient or their general medical practitioner. The role of the majority of oral healthcare providers is not necessarily to determine the diagnosis but to take appropriate actions when there is a possible abnormality, as this can, and often will, make a positive difference to the patient and indeed, those around them.

The aim of the present article is to provide a succinct review of the oral features of early systemic disease. There is no intention to discuss every possible scenario but to present the most likely and demonstrate the wide range of disease processes that may impact upon the mouth. The article does not consider the early orofacial features of systemic drug therapy, as while this is an area of immense change, it falls outside the scope of the present review.

## Key points

- The mouth is a major entry gate to the human body - it represents a mirror to overall health.
- Many diseases have mouth complications or even appear in the mouth before being diagnosed by general medical practitioners.
- Regular dental check-ups are important, not only to monitor dental health, but to also increase the likelihood that some systemic diseases - some of which may be significant - are diagnosed at earlier stages.

## Introduction

Like many other body structures, the mouth is often affected by disease that principally arises from, or targets, several tissue sites - a simple example being that acute myeloid leukaemia may, upon occasion, give rise to enlargement of the gingivae. There are many reviews of the oral manifestations of systemic disease, but there seems to be no succinct reviews of the early oral features of significant systemic disease. While not all individuals have readily available access to oral healthcare, a dentist, therapist, hygienist, or nurse may be the first to observe a feature of the mouth that sometimes reflects significant systemic disease unknown to the patient or their attending clinicians (usually their general medical practitioner). Oral disease can be the first, only, or become the most severe manifestation of a systemic disease and/or become the most significant factor to adversely affect an individual's quality of life.^1^

The aim of the present article is to provide a succinct review of the oral features of early systemic disease. The role of the majority of oral healthcare providers is not necessarily to determine the diagnosis, but to take appropriate actions when there is a possible abnormality, as this can, and often will, make a positive difference to the patient and indeed, those around them. For ease of presentation, the orofacial manifestations of early systemic disease will be considered on a site-by-site basis. There is no intention to discuss every possible scenario but to present the most likely and demonstrate the wide range of disease processes that can impact upon the mouth. The article does not consider the early orofacial features of systemic drug therapy, as while this is an area of immense change (e.g., medication-related osteonecrosis of the jaws and immune checkpoint inhibitor-induced sialadenitis and/or lichenoid-type disease),^2^^,^^3^ it falls outside the scope of the present paper.

## The lips

### Enlargement

Swelling of the lips is typically caused by local trauma but persistent swelling or swelling that repeatedly occurs may be secondary to unknown systemic disease.

Possibly the most likely cause of recurrent swelling of the lips is angioedema due to allergic disease.^4^ There can be a wide array of precipitants, and the swelling may sometimes, but not always, be accompanied by swelling of the face (for example, around the eyes), tongue, pharynx and larynx. Intermittent swelling may also arise as an initial feature of the rare C1-esterase inhibitor disorders and can be a rare feature of untoward, and sometimes early reaction of, angiotensin converting enzyme (ACE) inhibitors.^5^

**Fig. 1:**
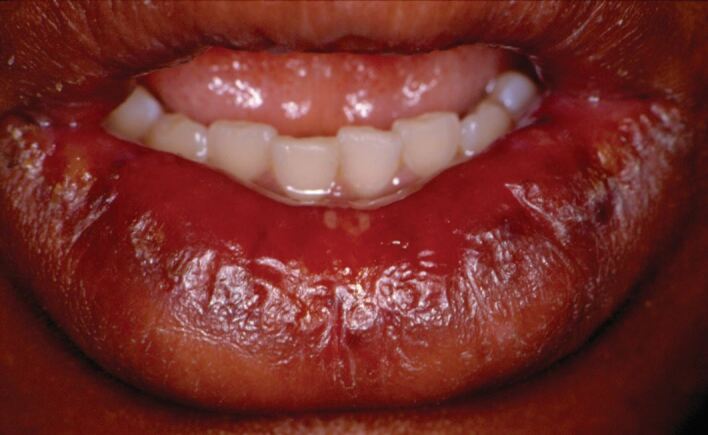
Labial enlargement of granulomatous disease, such as Crohn's disease, sarcoidosis, or orofacial granulomatosis

Persistent swelling of the lips can be an early feature of Crohn's disease.^6^ The swelling may affect one or both lips and can be unilateral or bilateral (Fig. 1). The persistent swelling may cause secondary angular cheilitis and median cheilitis is also possible. Sarcoidosis may also cause persistent lip swelling; although, the labial enlargement is probably not likely to be an early feature of this disorder - that may also give rise to salivary gland enlargement (usually the parotid glands) and/or a lower motor neuron palsy of the facial nerve akin to that of a Bell's palsy.^7^

### Redness

**Fig. 2:**
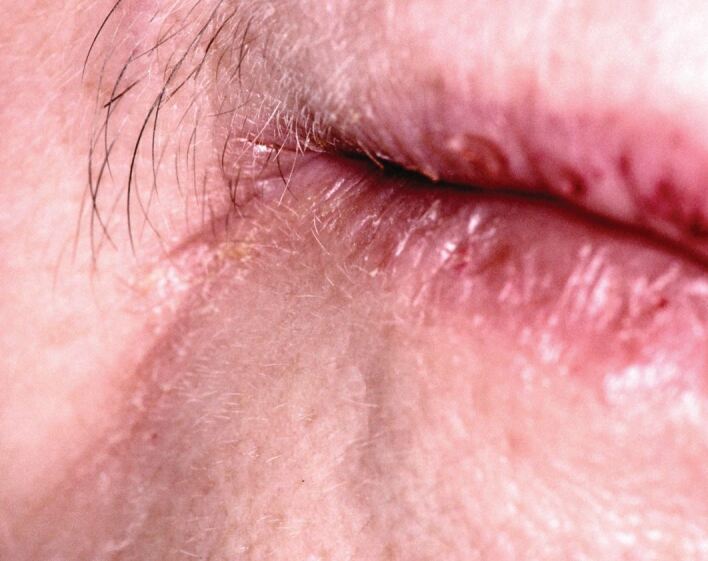
Angular cheilitis, typically caused by reduced vertical face height but also a feature of various anaemias and other systemic disorders

Redness of the complete lips is unusual (even polycythaemia does not seem to cause this, but erythema at the angles of the mouth is remarkably common). Angular cheilitis is redness, crusting and sometime ulceration at the corners of the mouth opening (Fig. 2). It usually reflects mild pooling of saliva at these sites that forms a nidus of infection, usually candida species with or without *Staphylococcus aureus* (of skin origin). This is most commonly due to reduced lip support/reduced lower vertical face height, secondary to loss of teeth, occlusal wear of dentures and, rarely, severe attrition.^4^ However, angular cheilitis may be an early feature of anaemia (typically a deficiency of iron, folate and/or vitamin B_12_ [the ‘haematinics']) that in turn may reflect a gamut of systemic disorders which often centre upon reduced uptake (e.g., gluten-sensitive enteropathy) or loss (e.g., inflammatory bowel disease and colonic/rectal cancers) of haematinics.^8^ A diet lacking in haematinics may also cause eventual anaemia and angular cheilitis, as may disorders of anaemia of other causes (for example, haematological malignancies). Often, angular cheilitis secondary to anaemia will be accompanied by redness of the dorsum of tongue (discussed below) and/or superficial mouth ulcers. Drooling (a loss of the ability of the saliva to be retained in the mouth - not hypersalivation, which is very rare) can give rise to notable angular cheilitis. Drooling can be an early feature of an upper motor neuron defect of the facial nerve (e.g., following a stroke) or lower motor neuron defect (for example, an acoustic neuroma [neuroma of the vestibulocochlear nerve], sarcoidosis and many others).^4^ In contrast, in Parkinson's disease, drooling is unlikely to be an early or presenting clinical feature.

### Other altered colour

The colour of lips varies between different races, but the lips can become blue as a consequence of central cyanosis due to, for example, cardiac septal defects (as in some individuals with Down syndrome), but this will not be an early presenting feature in patients attending for oral healthcare. Cyanosis of the lips can arise with anaphylaxis but clearly there will be much more significant signs of this potential medical emergency.

The lips have different degrees of hypermelanosis (brown colouration). This typically has a racial basis but hypermelanotic macules on and around the lips (‘peri-oral') can be an early sign of Peutz-Jeghers syndrome.^9^ Hypermelanotic pigmentation around the lips occur in 95% of affected individuals (who may often also have similar pigmented areas of the buccal mucosa).^9^ Aside from giving rise to anaemia and possible small bowel obstruction, Peutz-Jeghers syndrome can increase the risk of a spectrum of cancers, including those of the stomach, gastrointestinal tract, pancreas, breast, ovaries, uterus and testes. Thus, the early recognition of possible features of this, albeit uncommon, disorder has the potential to hasten diagnosis and possible treatment, ensure appropriate counselling of family members and lessen the risk of fatal disease.^10^

### Ulceration

Secondary herpes simplex virus (HSV) infection (usually HSV 1 or HSV 2) is probably the most common cause of lip ulceration. This usually presents repeatedly at the same area of the vermillion border progressing through phases of paraesthesia, erythema, vesiculation, pustule and later ulcer formation and eventual healing. This disorder may reflect exposure to sunlight, psychological distress or concommitant acute respiratory disease. However, a rapid increase in the frequency of such manifestations may be an indicator of underlying immunodeficiency.^11^

**Fig. 3:**
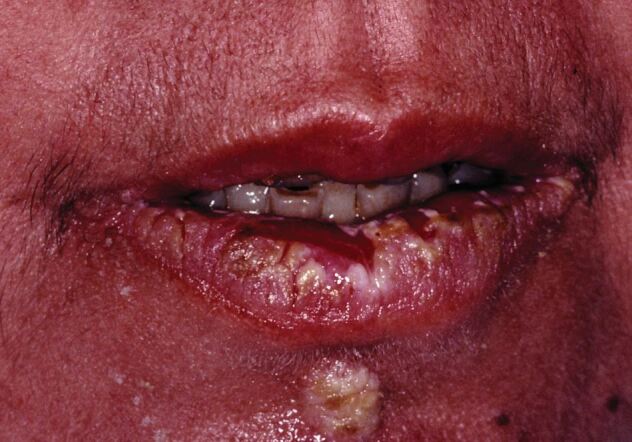
Labial ulceration secondary to pemphigus vulgaris (although the most common cause of ulceration of the lip area is possibly herpes simplex virus infection)

Much more clinically dramatic than herpes labialis, erythema multiforme (EM) can give rise to extensive superficial ulceration of both lips (usually on the inner labial mucosa) that may occasionally bleed and crust. While HSV may account for instances of EM-minor,^12^ EM usually arises as a consequence of concommitant drug therapy for a wide array of systemic disorders; although, it can be a feature of lung infection with *Mycoplasma pneumoniae* and sometimes other bacterial infections (when the term reactive infectious mucocutaneous eruption is considered appropriate).^13^ Other less common disorders that may initially manifest with labial ulceration include primary syphilis, mucous membrane pemphigoid and different types of pemphigus (Fig. 3). It is suggested that paraneoplastic pemphigus (now termed paraneoplastic autoimmune multi-organ syndrome) secondary to a host of, usually, haematological malignancies, will in particular give rise to multiple areas of ragged ulceration of the labial mucosae.^14^

## The gingivae and periodontium

### Gingival enlargement

Enlargement of the gingivae is almost always plaque-induced but there are instances when plaque-driven enlargement may be more profound than might be expected, or there is gingival enlargement that has no association with plaque. Examples of exaggerated gingival enlargement with an underlying systemic basis include drug-induced disease (e.g., calcium channel blockers, the calcineurin inhibitor ciclosporin and phenytoin),^15^ as well as disorders associated with a reduction in neutrophil number (e.g., the various congenital neutropenias) or function (e.g., chronic granulomatous disease).^16^ Such enlargements may not be an initial manifestation of underlying systemic disease but they can certainly be important in establishing the diagnosis of sometimes clinically significant problems.

Gingival enlargement may be the first sign of non-solid haematological malignancies, particularly acute myeloid leukaemia, and less commonly, chronic lymphocytic leukaemia, as well as non-Hodgkin's lymphoma.^17^ Metastatic disease from the lungs, colon, prostate and breast may manifest as localised gingival swellings; although, in most instances, this will not be the initial manifestation of the underlying malignancy.^18^ Before the advent of the almost universal availability of antiretroviral therapy (ART), Kaposi's sarcoma commonly manifested as a red, blue, or purple swelling of the gingivae and could thus be the initial features of unknown acquired immune deficiency syndrome.^16^

With regards to non-neoplastic disease granulomatosis with polyangiitis, it may cause unusual, usually localised gingival enlargement, which may include swelling of interdental papillae that have a strawberry-like appearance,^19^ while the even more rare hypoplasminogenaemia may manifest only as unusual deposits of fibrinous material on or around the gingivae.^20^ The sudden development of gingival bleeding may reflect an underlying, sometimes unknown, thrombocytopenia or acquired clotting disorder (for example, secondary to hepatic disease).^21^

### Pain and redness

**Fig. 4:**
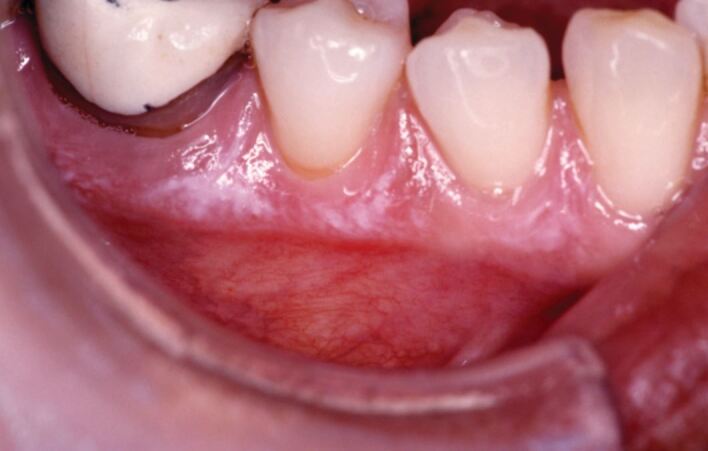
Lichen planus of the gingiva

Most plaque-induced gingival and/or periodontal disease is not painful. The most common reason for painful gingivae is likely to be desquamative gingivitis. This manifests as erythema without swelling of the attached and sometimes free gingivae that may have a localised or generalised distribution across the gingivae. Desquamative gingivitis is not a diagnosis - it is a descriptor. The actual cause is usually lichen planus (Fig. 4), and often desquamative gingivitis is the first manifestation of disease that may affect the skin and non-oral mucosal surfaces (typically the vulva or vagina). Other causes of desquamative gingivitis, in which the gingival disease may be the presenting signs, include mucous membrane pemphigoid and pemphigus vulgaris (and other variants); although, similar disease can present with dermatitis herpetiformis, linear IgA disease, lupus erythematosus (different types), mixed connective tissue disease and epidermolysis bullosa acquisita.^22^

### Exaggerated periodontal inflammation

As with plaque-induced gingival inflammation, periodontal disease can be worsened by systemic disease. This largely falls under three categories: defective immunity (usually reduced neutrophil number and/or function [the link with diabetes mellitus]), abnormal collagen structure (e.g., Ehler-Danlos syndrome) or defective cementum formation (e.g., X-linked 21-hydroxylase deficiency [vitamin D-resistant rickets]). Discussion of each of these is out of the scope of this article but it would seem prudent to consider that when the extent of periodontal inflammation is not in keeping with expectations, that consideration of an underlying systemic disease be given.

## The tongue

### Altered colour

**Fig. 5:**
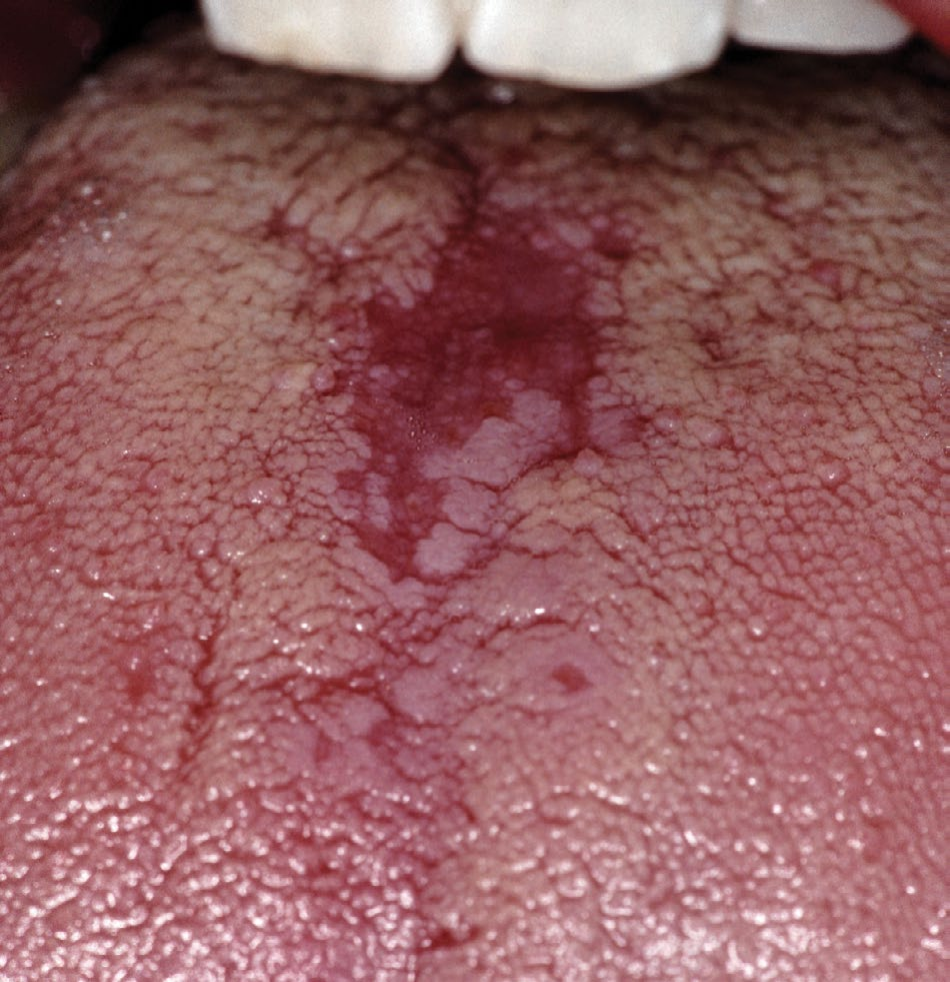
Chronic erythematous candidiasis (median rhomboid glossitis). In the primary care setting, this is probably most likely to be in patients who regularly use corticosteroid inhalers

A vast array of anomalies of the tongue are possible, some of which can be the initial manifestation of systemic disease. Central cyanosis (as discussed above) can give rise to a blue tongue, while methemoglobinaemia, secondary to dapsone therapy, may initially manifest as a blue-coloured tongue.^23^ Redness and flattening of the centre of the dorsum is usually an indication of candida infection (termed chronic erythematous candidiaisis, or median rhomboid glossitis) (Fig. 5), secondary to corticosteroid inhaler use or a prolonged dry mouth, but it can also be an early manifestation of an unknown underlying immunodeficiency, such as chronic mucocutaneous candidiaisis, diabetes mellitus, or even human immunodeficiency virus (HIV). Anaemias, as discussed previously, may cause a loss of the filiform papillae such that the tongue may take on a smooth and erythematous appearance called atrophic glossitis.^24^ White elevated patches on the lateral aspect of the tongue (termed oral hairy leukoplakia) may be the first manifestation of unknown immunodeficiency (usually HIV disease).^25^ Most of the disorders that give rise to hypermelanotic pigmentation of the buccal mucosae (see below) also affect the tongue in a similar manner. A rare form of hypermelanotic pigmentation that may affect the tongue - as patches of brown pigmentation - is Laugier-Hunziker syndrome (sometimes also termed ‘idiopathic lenticular mucocutaneous hyperpigmentation'). This is a sporadic, harmless disorder that arises in childhood in which there is also linear pigmentation of some nail plates (‘longitudinal melanonychia').^26^

### Swelling

Generalised swelling of the tongue can, as noted above, reflect angioedema of different causes but often seemingly unnoticed localised swellings can reflect remarkably significant disease, such as type 1 neurofibromatosis, multiple endocrine neoplasia type 2B, or amyloidosis (for example, secondary to multiple myeloma).^27^

### Abnormal movement

Abnormal tongue movements are not notably common but may be the initial feature of base-of-skull tumours (e.g., near or within the hypoglossal canal), manifesting as fasciculation or paralysis of one side. Bilateral fasciculation may be an early feature of certain types of motor neuron disease (e.g., amyotrophic lateral sclerosis and progressive bulbar palsy),^28^ or a late feature of Parkinson's disease. Tardive dyskinesia (within the newly classified ‘tardive syndrome') is possibly one of the more common movement disorders to include the tongue. However, this is generally considered to be a late onset feature (i.e., months to years) after exposure to the causative dopamine receptor blocking agents,^29^ usually with first- or second-generation antipsychotics. Of note is that occasionally, carbamazepine or metronidazole can give rise to facial dyskinesia.

## The palate

### Ulceration

Mucosal manifestations somewhat mirror those of the tongue and buccal mucosae (see below) but blistering (bullae) and/or ragged ulceration at the junction of the hard/soft palate can be the initial manifestation of later non-oral mucous membrane pemphigoid, pemphigus vulgaris, or epidermolysis bullosa acquisita. Superficial ulceration that sometimes (but actually rarely) is snake-like (serpiginous) or snail-track-like may reflect unknown secondary syphilis,^30^ while multiple superficial ulceration in a unilateral distribution is likely to be secondary herpes zoster (shingles), secondary to unknown or known immunodeficiency. Of note is that the palate may be the initial site of non-Hodgkin's lymphoma and/or Kaposi's sarcoma, also secondary to sometimes unknown immunodeficiency.^31^

## The buccal mucosae

Some of the aforementioned disorders can and commonly affect the buccal mucosae (for example, oral lichen planus [OLP]) thus the discussion will focus upon three signs: ulceration, swelling and pigmentation.

### Ulceration with or without white patches

In view of their predisposition to the traumatic effects of normal oral function, the buccal mucosae are common sites of ulceration caused by immunobullous disorders, particularly pemphigus vulgaris and mucous membrane pemphigoid. The latter may also give rise to blood-filled bullae, while the former, in view of the blister lining being thin, rarely presents as blistering and instead gives rise to ragged-edged superficial ulcers of varying size. As noted above, the mouth is a common initial site of lichen planus and the buccal mucosae are the most likely sites of oral involvement. OLP manifests as asymptomatic white-to-grey patches that may have a reticular (lace-like pattern), papular (multiple small papules) or plaque (large white areas) appearance. Within these, there may be areas of erythema (erythematous OLP), ulceration (ulcerative OLP), or very rarely, small blisters (bullous OLP).

The buccal mucosae are common sites of recurrent aphthous stomatitis (recurrent bouts of superficial oral ulceration of unknown cause arising in otherwise well persons). Although this has no association with systemic disease, similar types of ulceration can be an initial, and indeed are a cardinal feature of Behçet's disease,^32^ and also the autoinflammatory disorder PFAPA (periodic fever, aphthous stomatitis, pharyngitis, adenitis).^33^

Punched-out deep ulcers with a notable erythematous border can be an early feature of Crohn's disease. These can arise on almost any oral mucosal surface, but the buccal mucosae are a common site of involvement. The buccal vestibules are possibly the most common sites of these ulcers, often having a linear appearance with a rolled margin that may comprise small erythematous tag-like swellings. Patients may also have superficial ulceration due to a haematinic deficiency (usually iron and/or vitamin B_12_) caused by involvement of the terminal ileum or parts of the lower gastrointestinal tract.

### Swelling

**Fig. 6:**
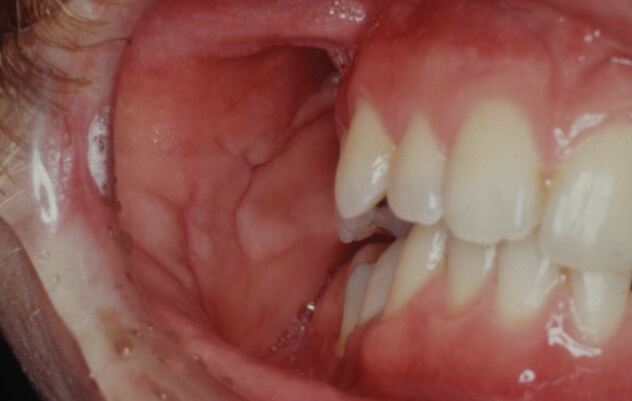
'Cobblestoning' of the buccal mucosa. This can be a feature of granulomatous disease (e.g., Crohn's disease)

Aside from giving rise to labial swelling and oral ulceration, unknown gastrointestinal Crohn's disease can cause diffuse swelling of the buccal (and sometimes labial mucosae) that has the appearance of a duvet (although is termed ‘cobblestoning') (Fig. 6). In addition, and independent of cobblestoning, there can be multiple collections of papular-like mucosal tags, particularly at the posterior aspects of the buccal mucosae.^6^

### Pigmentation

**Fig. 7:**
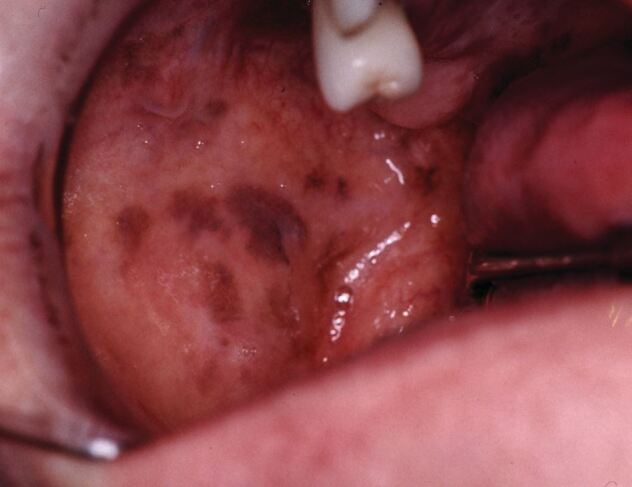
Hypermelanotic (brown) pigmentation of the buccal mucosa. This instance is physiological in cause; although, adrenocortical hypofuncton (e.g., Addison's disease) can give rise to this appearance

The buccal mucosae are a common site of racially associated (‘physiological') hypermelanotic pigmentation and the most common cause of abnormal pigmentation of the buccal mucosae is probably amalgam tattoo. However, they are also the most cited oral location of hypermelanotic pigmentation due to adrenocortical hypofunction (Fig. 7). Such underlying disease usually reflects autoimmune-driven destruction of the adrenal cortex (Addison's disease).^34^ Adrenocortical hypofunction often has an array of different and sometimes non-specific clinical manifestations; hence, the early recognition of possible oral pigmentation can greatly aid early definitive diagnosis of an important disorder.

## Altered sensation

### Pain

Most pain of the mouth reflects local causes and while it is often mentioned that pain of the left side of mandible may be a feature of angina pectoris or myocardial infarction, it is likely to be a comparatively minor feature of such disease. Some orofacial pain can be driven by, or at least associated with, psychological distress. A variety of orofacial pain disorders fall into this category, including burning mouth syndrome, atypical odontalgia, atypical facial pain and temporomandibular disorder. A description of these disorders is beyond the scope of this article, but the onset of such symptoms may reflect stressors that the affected person has no conscious awareness of.^35^^,^^36^

Orofacial pain can be due to neurological disease. Perhaps the most common instance of this is trigeminal neuralgia^35^, usually reflecting a demyelination of the trigeminal ganglion or root secondary to local pressure from an aberrantly located artery. Sometimes, however, symptoms identical to trigeminal neuralgia can be an early presentation of other causes of such demyelination, including multiple sclerosis and rarely, tumour (e.g, schwannoma, neurofibroma), inflammation (e.g., systemic lupus erythematosus), arterial aneurysms, arteriovenous malformations and even bony defects (e.g., osteopetrosis) within the middle cranial fossa.^37^

Pain of the mouth and face can occasionally reflect vascular disease, the most clinically significantly being giant cell arteritis. This can give rise to pain of the temple region of one side as well as a cramp-like pain of the masseteric region, sometimes termed masseteric claudication. Very occasionally, there can be necrotic (usually unilateral) ulceration of the tongue and/or lip, as walls of arteries (usually branches of the external or internal carotid) are inflamed and swollen. Early recognition of possible giant cell arteritis is important, as involvement of the retinal artery can ultimately lead to rapid onset loss of visual fields of one eye.^38^

### Paraesthesia/anaesthesia

Loss of sensation of touch, pain and temperature within the mouth is almost always due to physical trauma (e.g., fractures, iatrogenic damage); although less common instances include inappropriate placement of old-style endodontic materials and invasion by local malignancy or cysts (e.g., odontogenic keratocyst).^39^^,^^40^ However, trigeminal paraesthesia or anaesthesia can reflect an unidentified systemic disease. Metastatic spread of common malignancies, such as breast, lung and colon, as well as multiple myeloma (and others) can give rise to paraesthesia or anaesthesia of the lower lip. Similar symptoms can arise with demyelinating disease (e.g., multiple sclerosis), connective tissue disease (e.g., scleroderma and Sjogren's syndrome) and very occasionally, with longstanding diabetes mellitus and medication (e.g., some antimalarials).^41^^,^^42^ In most of these instances, patients with paraesthesia or anaesthesia of the lip or within the mouth will have systemic disease that is already known; however, rarely, there is no identifiable reason for this symptom, and yet it is a manifestation of unknown significant distant disease (particularly malignancy).

### Taste

Abnormalities of taste are most commonly linked to acute gingivitis, periodontitis and pericoronitis. Of course, dysgeusia, ageusia and phantogeusia were reported in as many as 60% or more of patients who were infected with the delta variant of SARS COV-2 and there remain individuals who are experiencing dysgeusia as an early feature of infection with the variants that are presently in populations. Taste disturbances can also arise as a side effect of a wide variety of medications (including metronidazole), or as a complication of systemic disease, such as Guillain-Barré syndrome, lesions of the skull base or cerebellopontine angle, cerebrovascular accident (e.g., stroke), neurodegenerative diseases (e.g., Parkinson's disease), multiple sclerosis, and cognitive impairment (Alzheimer's disease).^43^^,^^44^^,^^45^ As with altered pain sensation, the vast majority of individuals who have a taste abnormality caused by a non-oral factor will have systemic disease that is already known to them or those around them, but once in a while, the reason for the taste defect will not be evident, in which case referral by the oral healthcare provider to an appropriate service may allow a significant cause to be found.

## The salivary glands

Most disease that affects the salivary gland manifests as oral dryness and/or local swelling.

### Oral dryness

**Fig. 8:**
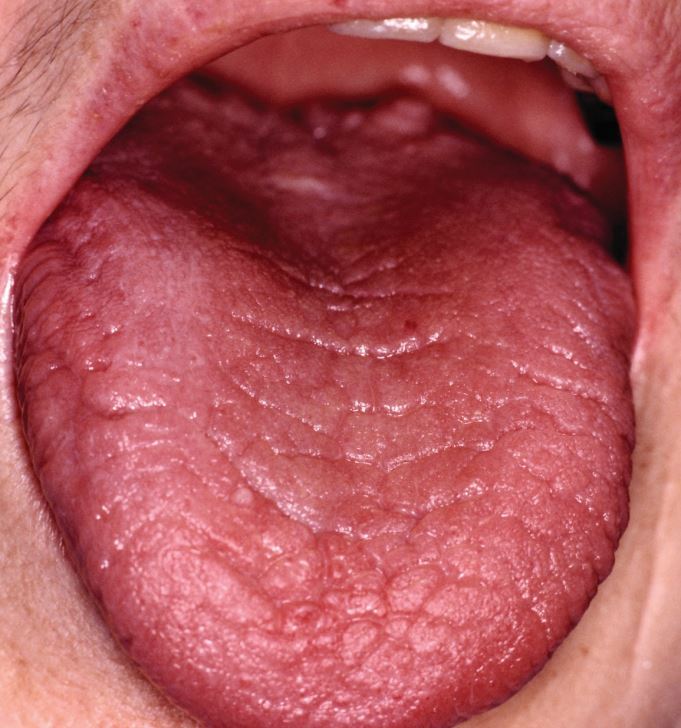
Lobulation and thinning of the tongue secondary to longstanding oral dryness, most likely to reflect long-term use of drugs with an anti-cholinergic action or Sjogren's syndrome

Prolonged oral dryness is often due to medication: typically, almost all groups of antidepressants and/or benzodiazepines (there are many others) (Fig. 8). However, oral dryness (the symptom is termed ‘xerostomia') is one of the predominant features of Sjogren's syndrome. This disorder causes autoimmune destruction of the salivary and lacrimal glands but also has a wide array of other features that can arise in almost any body structure. In addition, Sjogren's may accompany connective tissue disorders such as rheumatoid arthritis, systemic lupus erythematosus and others. The early diagnosis of Sjogren's syndrome is important as from an oral health perspective, affected persons are at risk of plaque-induced oral disease. Of note, patients with Sjogren's syndrome have an approximately 1-in-20 risk of developing non-Hodgkin's lymphoma in a major salivary gland, that, if diagnosed promptly, has a five-year-survival rate of up to 95%. In the past, before the advent of ART and directly acting anti-virals, HIV and hepatitis C infection could manifest with oral dryness and salivary gland swellings that potentially might be the first signs of underlying, significant, viral infection.

### Swelling

**Fig. 9:**
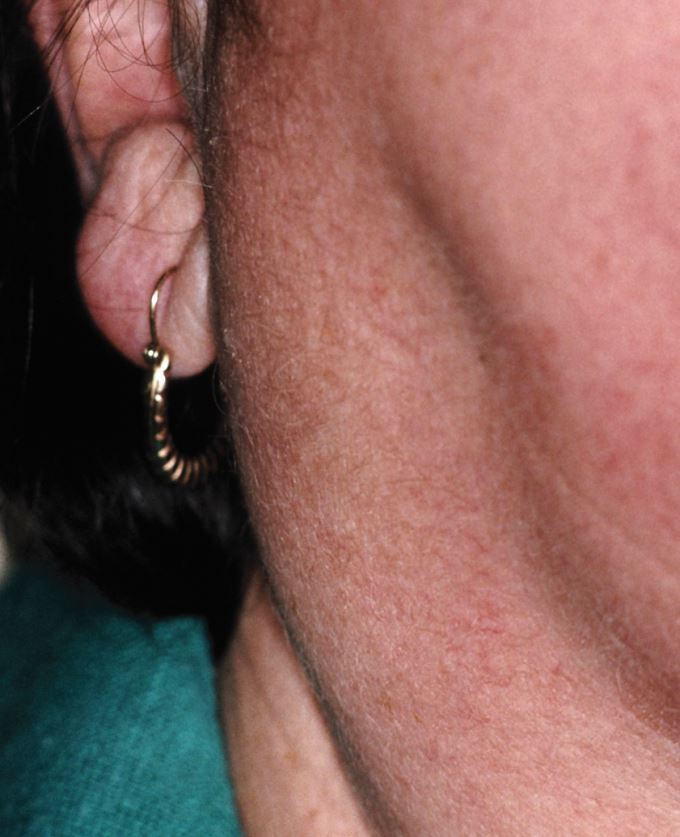
Mild out-turning of the pinna of the ear caused by parotid gland enlargement due to Sjogren's syndrome

Sialolithiaisis of the ductal network of a submandibular gland is probably the most common non-neoplastic cause for swelling of a major salivary gland but this does not have any consistent links with systemic disease. Swelling of one of both parotid glands together with symptoms or signs of prolonged oral dryness can be an early feature of Sjogren's syndrome (Fig. 9). Swelling of salivary glands of one or both sides can be an early feature of mumps, that unfortunately has returned due to the variable uptake of vaccination. Acute and chronic sarcoidosis can cause bilateral enlargement of the parotid glands and can, upon occasion, be the initial manifestation of this systemic disorder. Finally, a swelling within a single submandibular or parotid gland can be the first sign of IgG4-related disease, an uncommon disorder that can give rise to multisystemic disease but often with a focus upon the lacrimal glands and pancreas.^46^

## Conclusion

The mouth can be the initial, and sometimes only, site of involvement of systemic disease. A wide array of signs and symptoms are possible as a consequence of disease that ultimately will centre upon other body structures. Early recognition that something is amiss in or around the mouth, together with referral to an appropriate specialist, will, undoubtedly, hasten definitive diagnosis and effective management that, in turn, may maintain or improve an individual's wellbeing and quite possibly, duration of life.
